# SCAN gears up for high school

**DOI:** 10.1093/scan/nsy112

**Published:** 2019-01-04

**Authors:** Matthew D Lieberman

**Affiliations:** UCLA Psychology Department, 1248 Franz Hall, Los Angeles, CA, USA

In 2011, I wrote an editorial entitled `SCAN heads to kindergarten’ to highlight how *SCAN* had grown over its first 5 years of existence. My, how the time has flown and our little journal is gearing up for high school and soon will be asking for the keys to the car. When several of us first discussed starting a journal in 2004 and spent most of 2005 working on creating it, who could have imagined that 15 years and more than 1500 articles later that Social and Affective Neuroscience would be flourishing to the extent that few think of these fields as upstarts or novelties.

Just as in 2011, in 2019, *SCAN* is at an inflection point. For the first few years of the journal, I handled every single manuscript myself and was singularly focused on publishing new empirical neuroscience papers contributing to our understanding of social and affective processes in humans—an endeavor then in its infancy. Soon enough, it was clear to me that the field was expanding so quickly that I no longer had enough expertise to handle it all. Several new associate editors came on board to handle the different areas of social neuroscience. We also added our first non-empirical articles through our ‘Tools of the Trade’ and ‘In this Issue’ articles.

Now in 2019, more change is coming. The editorial board (i.e. consulting editors) has been largely constant since 2005 when the board was first created. Many on the board were senior faculty who were *friends* of social and affective neuroscience without doing it themselves or perhaps collaborating occasionally with folks who did. This was essential early on because it gave credibility to a budding field that had little at the time. I am grateful to everyone who has served as consulting editor and has offered their advice and support over the past 14 years.

But now the field has moved on. Where once I and a few others were referred to as ‘young Turks’ at the start of things, we are now the ‘greying Turks’. Happily, we are increasingly supplanted by hundreds of amazing young social and affective neuroscience doing groundbreaking research, taking our science forward into a new era. It is time to start giving the editorial board over to them so they can offer their advice (and hopefully support) on what *SCAN* should look like going forward.

Almost all of the consulting editors are new and the vast majority are assistant or associate professors. Our associate editors each nominated multiple consulting editors and I added several invitations of my own. The resulting crop is 56% female and 32% are at non-US universities. We are happy not only about what this diversity says about *SCAN* but what it says about the state of the field. I fully expect that many of the future associate editors to come from this list of new consulting editors.

A second change is that we are now actively requesting submissions of review papers, meta-analyses and papers based on large-scale databases (e.g. Neurosynth). When the journal started, I specifically opposed having non-empirical papers because I did not want *SCAN* to be full of wildly speculative papers. But now we have so much data in social and affective neuroscience, that some of the most exciting work involves integrating the existing data into larger frameworks and analyses. Ideally, we will have one of these types of papers per issue and they would appear at the beginning of the issue in most cases. While many review papers can be successfully fit within our long-standing 5000-word limit, some really need to be longer, and thus, review papers can be up to 10,000 words. We will be highly selective in the reviews that we take. A successful review should make a point or argument that has not already been made. A new meta-analysis on a topic that has received recent quality meta-analytic attention will be unlikely to be successful at SCAN. Finally, to facilitate high-quality consideration of meta-analyses, SCAN has its first Associate Editor specializing in meta-analyses (Hedy Kober).

The final change to *SCAN* is that we are going to increase the emphasis on special issues. Long ago, edited books played a very important role in presenting the field with a coherent summary of the current state of an area of research. Some of these books were highly influential. However, these days a high-quality special issue is more likely to serve the goals that were once served by edited books and do so better and faster than edited books ever could. Because *SCAN* is open access, everyone has immediate full access to all the articles in each special issue. Additionally, for the foreseeable future, all contributions to special issues will have their open access fee waived.

We have several exciting special issues lined up including: Computational Methods in Social Neuroscience (edited by Carolyn Parkinson), Health Neuroscience (Tristen Inagaki), Neuroforecasting (Brian Knutson and Carolyn Yoon), Clinical Neuroscience (Hedy Kober), Social Reinforcement (Andreas Olsson), Near-Infrared Spectroscopy in Social and Affective Neuroscience (Shannon Burns and myself), Social Neuroscience Meets Network Neuroscience (Lucina Uddin) and Communications Neuroscience (Emily Falk).

If you have an idea for a special issue, contact me and I will give you all the background information you need to think about when putting together a proposal. Proposals will be considered by the associate editors and myself. We will decide which proposals will go forward and also how they can be improved to create the highest-impact special issues possible. In addition to being a great service to the field, serving as a guest associate editor can give someone who has not yet done this a taste of what is involved in serving as an associate editor.

So there you have it. *SCAN* is headed to high school. It has its backpack, notebook and milk money all ready to go. We look forward to all of you contributing to *SCAN*, helping to keep it an essential node in the fields of social and affective neuroscience.

